# *In ovo* feeding of creatine pyruvate alters energy metabolism in muscle of embryos and post-hatch broilers

**DOI:** 10.5713/ajas.18.0588

**Published:** 2019-01-02

**Authors:** Tong Yang, Minmeng Zhao, Jiaolong Li, Lin Zhang, Yun Jiang, Guanghong Zhou, Feng Gao

**Affiliations:** 1 Jiangsu Key Laboratory of Animal Origin Food Production and Safety Guarantee, College of Animal Science and Technology, Nanjing Agricultural University, Nanjing 210095, China; 2 Jiangsu Collaborative Innovation Center of Meat Production and Processing, Quality and Safety Control, Nanjing Agricultural University, Nanjing 210095, China; 3 Ginling College, Nanjing Normal University, Nanjing 210097, China

**Keywords:** Broilers, *In ovo* Injection, Creatine Pyruvate, Muscle, Energy Reserves

## Abstract

**Objective:**

This study was conducted to investigate the effects of *in ovo* feeding (IOF) of creatine pyruvate (CrPyr) on the energy metabolism in thigh muscle of embryos and neonatal broilers.

**Methods:**

A total of 960 eggs were randomly assigned to three treatments: i) non-injected control group, ii) saline group injected with 0.6 mL of physiological saline (0.75%), and iii) CrPyr group injected with 0.6 mL of physiological saline (0.75%) containing 12 mg CrPyr/egg on 17.5 d of incubation. After hatching, 120 male chicks (close to the average body weight of the pooled group) in each group were randomly assigned to eight replications. The feeding experiment lasted 7 days.

**Results:**

The results showed that IOF of CrPyr increased glucose concentrations in the thigh muscle of broilers on 2 d after injection (p<0.05). Compared with the control and saline groups, the concentration of creatine in CrPyr group was increased on 2 d after injection and the day of hatch (p<0.05). Moreover, IOF of CrPyr increased the creatine kinase activity at hatch and increased the activities of hexokinase and pyruvate kinase on 2 d after injection and the day of hatch (p<0.05). Chicks in CrPyr group showed higher mRNA expressions of glucose transporter 3 (GLUT3) and GLUT8 on the day of hatch (p<0.05).

**Conclusion:**

These results demonstrated that IOF of CrPyr was beneficial to enhance muscle energy reserves of embryos and hatchlings.

## INTRODUCTION

During the last phase of avian embryogenesis, in order to satisfy the enormous energy requirement for hatching process, nutrients in the embryo are dramatically depleted [1]. Due to the limitation of oxygen availability, energy metabolism switches from lipid metabolism to glycogen metabolism in embryos [2,3]. As the predominant energy source at the end of incubation, the glycogen storage is significantly exhausted. Moreover, since the deficiency of carbohydrates makes the embryo select muscle fibers with type IIb for mobilization, the gluconeogenesis will de-plete muscle protein and have disadvantageous effect on muscle development [4]. In addition, under commercial poultry production the early hatching chicks are generally without feed for 36 to 72 h because of the variation of hatching process, chick handling and transportation [5,6]. Insufficient energy during this period impairs broilers growth irreversibly. Previous research reported that the irreversible damage could affect subsequent growth performance of chicks through to marketing age [7]. Therefore, supplying a stable and sufficient energy status in late-term embryogenesis is important for facilitating the muscle growth of broilers.

Some studies have been done to enhance the early nutrition of poultry. *In ovo* feeding (IOF), a method of early nutrition technology injecting exogenous nutrient solutions into the amniotic fluid of embryo, has been considered to effectively improve the energy reserves of the late-term embryos [8]. As mentioned in the previous literature, IOF of exogenous nutrient substances could enhance energy status, intestinal function and growth performance of poultry [9,10,11]. Creatine (Cr), the endogenous synthesis of glycine, arginine and methionine, which can be phosphorylated as phosphocreatine (PCr), is a nitrogenous molecule for energy metabolism [12,13]. The Cr/PCr system plays an important role in maintaining energy homoeostasis that can transfer a phosphate group to adenosine diphosphate (ADP) to resynthesis adenosine triphosphate (ATP) [12,14]. Pyruvate is an intermediate metabolite of carbohydrate and protein metabolism, which is crucial for energy metabolism by glycometabolism and the Krebs cycle. Pyruvate can be bound to creatine to enhance the use of both compounds (creatine pyruvate, CrPyr) [15]. Crpyr has both pyruvate and creatine functions and is more easily absorbed in the body. In addition, CrPyr has been reported to be more effective than pyruvate or creatine alone on energy metabolism [15,16]. A preliminary experiment in our laboratory has indicated that IOF of 12 mg/egg CrPyr increased hatching weight of male chicks, body weight (BW) gain and feed intake during 8 to 21 days but had no significant effect on feed/gain ratio [11]. In addition, previous research reported that 12 mg/egg CrPyr did not affect the hatchability of chicks, implying that the injection dose of CrPyr in research was safe and feasible [11]. Based on these findings, the objective of this research was to examine whether the regulation of energy metabolism affects growth performance by IOF of CrPyr in late-term incubation.

Hence, this study was to investigate the effects of IOF of CrPyr on muscle energy reserves, enzyme activities and mRNA expressions of energy metabolism in the thigh muscle of broilers from 19 d of incubation to 7 d post-hatch.

## MATERIALS AND METHODS

### Egg incubation

All animal care procedures in this experiment were approved by the Institutional Animal Care and Use Committee of Nanjing Agricultural University. A total of 1,200 fertile broiler eggs (Arbor Acres) were selected and purchased from Hewei Agricultural Development Co. Ltd. (Xuancheng, China). All eggs were weighed, and their average weight was 69.85 g (ranged from 68 to 72 g). Then, the eggs were randomly transferred to a microcomputer automatic incubator (ZCA-A, Zhicheng Incubation Equipment Co. Ltd., Dezhou, China). The temperature and relative humidity of the incubator was set at 37.8°C ±0.1°C and 60%. On embryonic day 6 and 16, the eggs were candled for discarding unfertilized and nonviable eggs. Afterwards, 960 eggs with similar weight close to the average weight (64.50±0.38 g) of the remaining were randomly distributed to one of three treatments and each treatment contained 8 replications of 40 eggs each.

### *In ovo* injection procedure

The 3 treatment groups included: i) non-injected control treatment, ii) saline treatment injected with 0.6 mL of 0.75% physiological saline solution, and iii) CrPyr (Hubei Ju sheng Technology Co. Ltd., Wuhan, China) treatment injected with 0.6 mL of 0.75% physiological saline solution containing 12 mg CrPyr/egg. This optimal concentration of CrPyr injection solution was selected based on the preliminary experiment in our lab [11].

All injection solutions were freshly prepared on 17.5 d of incubation. The CrPyr was dissolved in 0.75% NaCl diluents solution to a concentration of 20 mg/mL. Then, solutions were sterilized by filtration through a 0.22-μm membrane filter and subsequently kept in the incubator at 37.8°C for 2 h prior to administration. The eggs of Saline and CrPyr groups were candled to identify the location of amnion after the injection site of the egg was disinfected with 0.1% bromogeramine solution [8]. A hole was punched using a needle at the top of eggshell, and the solution was injected into the amnion by a 21-gauge needle [9]. The holes were sealed with paraffin after the injection, then the eggs were returned immediately to the hatching trays. The whole injection experiment process was completed within 2 h. Although the control group were non-injected, it was subjected to the same handling procedures as the other treatment groups.

### Animal feeding experiment

On the day of hatch, all male hatched chicks from one treatment were pooled and weighed. A total of 120 healthy male chicks from each of the 3 treatments with similar weights close to the average BW of their pooled group were randomly assigned into 8 replicates with 15 birds each replicate. Then, all the birds were reared in a temperature-controlled room and under incandescent white light with a light schedule of 23 h light and 1 h dark. The chickens were given free access to feed and water in three-layer cages, and the diet was formulated to meet the nutrient requirements of poultry (Table 1). The temperature in the chicken house was maintained at 32°C to 34°C for the first three days and then gradually reduced to 30°C until the 7 d of age.

### Sample collection

Two days after the injection, entire male embryos that were randomly selected by observing the morphology of the gonads were removed and cleaned of the yolk sac and membrane after opening the eggs at the air chamber. Samples were collected based on a modified method as described by Zhao et al [11]. Next, one embryo that was randomly selected from each replicate with a BW close to the average BW of the replicate, was euthanized with sodium pentobarbital (20 mg/kg of BW; Beijing Chemical Co, Beijing, China). Then, the entire left thigh muscle tissue from the embryo was collected and frozen in liquid nitrogen after weighed and recorded.

One bird per replication with a BW close to the average BW of the replicate was selected and weighed on the age of hatch, 3 and 7 d post-hatch. The entire left thigh muscle of the chicken was obtained and weighed after cervical dislocation, and the muscle tissue was collected in RNAase-free tubes, frozen in liquid nitrogen for further analysis.

### Analysis of pyruvate, glycogen, glucose, and lactic acid concentrations

The concentration of pyruvate, glucose, and lactic acid in the thigh muscle were determined according to the recommended procedures of a commercially available kit from Nanjing Jiancheng Bioengineering Institute (Nanjing, China). The glucose concentrations in the thigh muscle was measured by the commercially available kit from Shanghai RongSheng Biotech Co. Ltd. (Shanghai, China), based on the glucose oxidase method.

### Analysis of creatine and phosphocreatine concentrations

The concentrations of Cr and PCr in the thigh muscle were determined by high performance liquid chromatography (HPLC), which was modified from the method of Li et al [17]. Approximately 300 mg of each frozen muscle sample was accurately weighted and homogenized for 1 min in 2 mL of ice-cold 5% HClO_4_. After incubation in an ice bath for 15 min, the homogenate was centrifuged for 10 min at 10,000×g at 4°C. Then, the supernatant was mixed with 900 μL of 0.8 M K_2_CO_3_. After neutralizing in an ice bath for 10 min, the homogenate was centrifuged at 10,000×g, 4°C for additional 10 min. The supernatant (1.5 mL) was filtered through a 0.45-μm membrane filter, and 20 μL of the sample was injected into a Waters Alliance HPLC system 2695 (Waters, Milford, MA, USA) equipped with an auto sampler. Chromatographic separation was performed on a Waters SunFire C18 column (250 mm×4.6 mm, 5 μm) with a column temperature of 25°C. UV detection was performed at the wavelength of 210 nm. The mobile phase was made up of methyl cyanide and 29.4 mM KH_2_PO_4_ buffer (methyl cyanide: KH_2_PO_4_ = 2:98; v/v). The flow rate was set at 1.0 mL/min.

### Analysis of hexokinase, pyruvate kinase and creatine kinase activities

Earch frozen muscle sample (0.5 g) was weighed and homogenized in a centrifuge tube with 4.5 mL 0.75% saline and then centrifuged at 3,500×g for 10 min at 4°C. The supernatant was used for assaying the activities of hexokinase (HK), pyruvate kinase (PK), and creatine kinase (CK) with commercial kits (Nanjing Jiancheng Bioengineering Institute, China). As described by Panserat et al [18], the HK activity is based on the coupling ribulose-5-phosphate formation from glucose 6- phosphate to the reduction of nicotinamide adenine dinucleotide phosphate. The detec-tion principle of PK activity is based on the decreased rate of nicotinamide adenine dinucleotide hydrate during the conversion of phosphoenol pyruvate into pyruvate, and the conversion of pyruvate into lactate [19]. As described by Zhao et al [11], the result of CK was normalized against total protein concentration in each sample and the concentrations of protein in tissue extracts were estimated based on the manufacturer’s protocol quantitative assay kits (Nanjing Jiancheng Bioengineering Institute, China).

### Total RNA extraction and real-time polymerase chain reaction

Total RNA of thigh muscle samples was extracted using RNAiso Plus (Takara Biotechnology Co. Ltd., Dalian, China) according to the manufacturer’s instructions. The purity of the total RNA was evaluated according to the ratio of 260/280 using a spectrophotometer (Thermo Fisher Scientific, Wilmington, DE, USA). The RNA with the ratios between 1.9 and 2.0 were used for subsequent reverse transcription to cDNA using a PrimeScriptTM RT Master Mix kit (Takara Biotechnology Co. Ltd., China). The mRNA expression of the primer was evaluated using Real-time quantitative polymerase chain reaction (PCR). Reactions of real-time quantitative PCR analysis were performed using ABI 7500 Real-time PCR detection system (Applied Biosystems, Foster City, CA, USA), and the reaction system including: 10 μL of SYBR Premix Ex Taq (2×), 7.8 μL of RNA enzyme-free water, 0.4 μL of ROX Reference, 1 μL of cDNA, 0.4 μL of forward primers, 0.4 μL of reverse primers. The following process was administrated by steps at 95°C for 30 s, with 40 cycles at 95°C for 5s and 60°C for 30 s, then 95°C for 15 s, 60°C for 1 min and 95°C for 15 s. The results of relative mRNA expression level were calculated using the 2^−ΔΔCT^ method [20]. The primes sequences designed in GenBank were synthesized by Sangon Biotechnology (Shanghai, China) (Table 2).

### Statistical analysis

The data analysis was performed by one-way analysis of variance using IBM SPSS Statistics 20 software. Differences among treatments were examined using Tukey’s multiple range tests. The differences were considered to be significant at p<0.05.

## RESULTS

### Concentrations of pyruvate, glycogen, glucose, and lactic acid

The results of IOF of CrPyr on pyruvate, glycogen, glucose, and lactic acid concentrations in the thigh muscle are presented in Table 3 and 4, respectively. No difference in glycogen and lactic acid was observed among treatments (p>0.05). The glucose concentration in the muscle of CrPyr group was higher than that of the other treatments on the day of hatch (p<0.05).

### Concentrations of creatine and phosphocreatine

As shown in Table 5, IOF of CrPyr increased the Cr concentration compared to the other groups on 2 d after injection and on the day of hatch (p<0.05). No significant difference was observed in PCr concentration among all the groups at any of the measured time points (p>0.05).

### Hexokinase, pyruvate kinase and creatine kinase activities

The results of IOF of CrPyr on HK, PK, and CK activities are presented in Table 6 and 7. Compared with the control and saline groups, a significant increase in activity of CK was observed in CrPyr treatment on 2 d after injection, the day of hatch and 3 d post-hatch (p<0.05). Additionally, the activity of HK of IOF of CrPyr group was higher than other treatments on 2 d after injection and the activity of PK of IOF of CrPyr group was higher than other treatments on 2 d after injection, the day of hatch and 3 d post-hatch (p<0.05).

### Glucose transporters mRNA expressions

As shown in Table 8, the glucose transporter 3 (GLUT3) mRNA expression of IOF of CrPyr group was significantly higher than that of other groups on the day of hatch (p<0.05). Similarly, The GLUT8 mRNA expression of IOF of CrPyr group was also higher than that of other groups on the day of hatch (p< 0.05).

### Glycogen synthase and glycogen phosphorylase mRNA expressions

The results of IOF of CrPyr on glycogen synthase and glycogen phosphorylase mRNA expressions in the thigh muscle are shown in Table 9. No significant difference was observed in glycogen synthase and glycogen phosphorylase mRNA expressions among all the groups at any of the measured time points (p>0.05).

## DISCUSSION

Due to the limited oxygen during the last period of incubation, chick embryos preferentially use glucose as the energy source compared to lipids and proteins [2]. Gluconeogenesis becomes the critical source of glucose. In the avian embryo, the primary gluconeogenic precursors are amino acids, derived from the amnion and muscles [21,22]. Many researches have demonstrated that supplementation with exogenous nutrients via IOF technology could improve energy reserves during late incubation of embryos and neonatal broilers [1]. Wang et al [23] reported that dietary creatine monohydrate (1,200 mg/kg for 14 days) can elevate Cr concentration in both pectoralis major and tibialis anterior muscles. In skeletal muscles, Cr/PCr system is important to maintain energy balance. The Cr can be phosphorylated into PCr, which is directly related to the energy buffer system in skeletal muscle [12]. When ATP is depleted, the CK transfers a phosphate group from PCr to ADP to regenerate ATP and maintain energy homeostasis in the muscle [13]. The current study indicated that IOF of 12 mg CrPyr/egg increased the Cr concentration in thigh muscle on 2 d after injection and the day of hatch but did not affect the PCr concentration at any of the measured time points, which is not consistent with the previous study in our lab that IOF of creatine monohydrate during the late phase of avian embryonic period increased both Cr and PCr concentrations in the breast muscle [10]. Breast muscle consists of type IIb fibers, whereas the thigh muscle consists of type I, type IIa, and type IIb fibers [24]. Different muscle fiber types have differences in creatine metabolism. When oxygen is deficient during last stage of incubation, muscle could utilize exogenous Cr to generate more PCr for ATP synthesis. In the present study, the Cr/Pcr system may serve as an additional energy source for muscle development in the CrPyr group, thereby improving the energy status of embryos during the last stage of incubation.

It has been noted that skeletal muscle requires GLUTs for the uptake of glucose from blood. Therefore, GLUTs play a vital role in maintaining glucose homeostasis. Unlike mammals, it’s reported that broiler chickens lack the GLUT isoform 4, but have the GLUT1, 3, and 8 [25–27]. Ju et al [26] reported that supplementation of creatine in rat diets increased phosphorylation of AMPK protein and increased mRNA expression of GLUT4 in pectoral muscle. Similarly, the present study showed that IOF of CrPyr significantly increased GLUT3 mRNA expression on the day of hatch in comparison to other groups. The results indicated that the muscle cells increased the capacity to uptake glucose.

As expected, the results showed that IOF of 12 mg CrPyr/egg increased the concentration of glucose at hatch. The late term embryo orally consumes the amniotic liquid before hatching and therefore the IOF administered CrPyr is presented to the enteric tissues for digestion and absorption and then can be utilized or converted to glucose [28]. Moreover, previous study indicated that IOF of nutrients increased the activity of liver glucose-6-phosphatase enzyme and promoted hepatic gluconeogenesis [3]. In the present study, we speculated CrPyr could supply pyruvate as a potential substrate for hepatic gluconeogenesis. Once glucose enters the muscle, it is immediately converted to glucose-6-phosphate catalyzed by HK, and the glucose-6-phosphate is routed into the major pathways of either glycogenesis as glycogen or glycolysis as power [27,29]. But there was no significant change in glycogen concentration of thigh muscle in this research, which is consistent with the results of Foye et al [30], who found that muscle glycogen reserves in turkeys were not affected by IOF of 0.1% β-hydroxy-β-methylbutyrate with or without 0.7% Arg. Similarly, Tangara et al [31] reported that exogenous nutrients had no influence on muscle glycogen level in ducks. However, some research showed that IOF of carbohydrates improved the muscle glycogen level [9]. The difference among these findings may be explained by whether IOF of exogenous nutrients can stimulate insulin release. It’s demonstrated that insulin can promote dephosphorylation and activation of glycogen synthase by inactivating the glycogen synthase kinase 3 [32,33]. Previous research showed that IOF of carbohydrates could enhance the content of the glycogen in the muscles mainly by stimulating the release of insulin [30]. Therefore, IOF of CrPyr, a non-carbohydrate nutrient, had no effect on muscle glycogen level, which might be associated with lack of the insulin secretion. Furthermore, the present research showed that IOF of 12 mg CrPyr/egg had no effect on glycogen synthase mRNA expression as well as the expression of glycogen phosphorylase mRNA in broiler muscle, which further proved that glucose uptake in the muscle is not synthesized as glycogen stored in muscles.

At the end of the egg incubation period, glycolysis becomes the predominant energy source of avian embryo and neonates [1,2]. There are three key enzymes of glycolysis, namely, HK, PK, and phosphofructokinase (PFK). In the present study, IOF of 12 mg CrPyr/egg significantly enhanced HK and PFK enzyme activities on the day of hatch, which suggests that exogenous CrPyr stimulated the glycolysis process and it may have a positive effect on the energy supply of broiler muscle during hatching. In this research, the pyruvate and lactic acid levels in muscle were not altered significantly. During the glycolysis process, pyruvate as an energy metabolism intermediate can turn into lactic acid, which is then converted back to glucose in the liver by Krebs cycle.

Conclusion can be drawn from the present study that IOF of 12 mg CrPyr/egg at 17.5 d of incubation could increase the Cr concentration in the thigh muscle on 2 d after injection and the day of hatch, and the activity of CK in the thigh muscle on 2 d after injection, the day of hatch and 3 d post-hatch, which was beneficial for improving the energy buffer system in the muscles of broilers. In addition, injection of CrPyr increased glucose concentration in the thigh muscle possibly through the up-regulation of GLUTs mRNA expressions, which may be useful to enhance energy status of embryos and hatchlings. Therefore, IOF of CrPyr could be an effective approach for enhancing early nutrient supply by altering the energy metabolism of broilers.

## Figures and Tables

**Table 1:** The composition and nutrient levels of basal diets

Items	Value
Ingredients (%)	
Corn	57.61
Soybean meal	31.00
Corn gluten meal	3.29
Soybean oil	3.11
Limestone	1.20
Dicalcium phosphate	2.00
L-lysine	0.34
DL-methionine	0.15
Salt	0.30
Premix1)	1.00
Calculated nutrient levels	
ME (MJ/kg)	12.56
CP (%)	21.10
Ca (%)	1.00
Available phosphorus (%)	0.46
Lysine (%)	1.20
Methionine (%)	0.50
Methionine+cysteine (%)	0.85

ME, metabolizable energy; CP, crude protein.

1) Premix provided per kilogram of diet: retinyl acetate for vitamin A, 12,000 IU; cholecalciferol for vitamin D_3_, 2,500 IU; DL-α-tocopheryl acetate for vitamin E, 20 IU; menadione sodium bisulphate, 1.3 mg; thiamin, 2.2 mg; riboflavin, 8.0 mg; nicotinamide, 40 mg; choline chloride, 400 mg; calcium pantothenate, 10 mg; pyridoxine·HCl, 4 mg; biotin, 0.04 mg; folic acid, 1 mg; vitamin B_12_ (cobalamin), 0.013 mg; Fe (from ferrous sulfate), 80 mg; Cu (from copper sulphate), 8.0 mg; Mn (from manganese sulphate), 110 mg; Zn (from zinc sulfate), 60 mg; I (from calcium iodate), 1.1 mg; Se (from sodium selenite), 0.3 mg.

**Table 2:** The primer sequences for real-time quantitative polymerase chain reaction analysis

Genes	Primer sequences (5′→3′)	Product size (bp)	GeneBank Identification
*GAPDH*	F:GAGGGTAGTGAAGGCTGCTG R:CATCAAAGGTGGAGGAATGG	113	NM_204305
*GLUT3*	F:ATGCTCTTCCCCTATGCTGA R:AAAAGTCCTGCCCTTGGTCT	123	NM_205511
*GLUT8*	F:GCAAGGGGTGTATCAAGCAG R:GGCAGAGAAGAGCCAGAATG	126	NM_204375
*PYG*	F:CTTTGGGATGAGGGTGGAG R:ATCTGGTCAACTGCCTGCTT	105	NM_204392.1
*GYS2*	F:ATCGCCTTCTGTCTCTCAGC R:TTTTGCCTATCCCTTTCAGC	108	XM_015291547.1

*GAPDH*, glyceraldehyde-3-phosphate dehydrogenase; *GLUT3*, glucose transporter 3; *GLUT8*, glucose transporter 8; *PYG*, glycogen phosphorylase; *GYS2*, glycogen synthase.

**Table 3:** Effects of *in ovo* feeding of creatine pyruvate on the concentration of glycogen and glucose in thigh muscle of embryos and broilers on 2 d after injection, the day of hatch, 3, and 7 d post-hatch

Items	Treatment1)			SEM2)	p-value

	Control	Saline	CrPyr		
Glycogen (mg/g)					
2 d after injection	1.33	1.35	1.36	0.02	0.816
Hatch	1.07	1.09	1.09	0.01	0.866
3 d	1.55	1.75	1.76	0.07	0.339
7 d	0.88	0.82	0.92	0.02	0.067
Glucose (mg/g)					
2 d after injection	1.33^b^	1.35^b^	1.49^a^	0.03	0.047
Hatch	1.47	1.53	1.59	0.05	0.631
3 d	1.53	1.64	1.66	0.03	0.111
7 d	1.52	1.42	1.43	0.03	0.208

The results are presented by mean values and the SEM.

CrPyr, creatine pyruvate; SEM, standard error of the mean.

1) Control, non-injected control group; Saline, saline control group with 0.6 mL physiologicalsaline (0.75%) per egg; CrPyr, CrPyr treatment group with 0.6 mL physiological saline (0.75%) containing 12 mg CrPyr per egg.

2) Pooled SEM (n = 8).

a,b Means within a row with different superscripts are different at p<0.05.

**Table 4:** Effects of in ovo feeding of creatine pyruvate on the concentration of pyruvate and lactic acid in thigh muscle of embryos and broilers on 2 d after injection, the day of hatch, 3, and 7 d post-hatch

Items	Treatment1)			SEM2)	p-value

	Control	Saline	CrPyr		
Pyruvate (nmoL/mg protein)					
2 d after injection	11.83	12.53	14.02	0.52	0.247
Hatch	13.49	14.25	13.43	0.35	0.587
3 d	11.96	11.46	11.81	0.22	0.663
7 d	7.39	7.11	7.58	0.14	0.529
Lactic acid (mg/g)					
2 d after injection	11.46	12.12	12.65	0.38	0.510
Hatch	13.07	15.41	15.61	0.62	0.175
3 d	13.60	13.64	14.61	0.37	0.508
7 d	10.28	10.51	10.79	0.14	0.707

The results are presented by mean values and the SEM.

CrPyr, creatine pyruvate; SEM, standard error of the mean.

1) Control, non-injected control group; Saline, saline control group with 0.6 mL physiologicalsaline (0.75%) per egg; CrPyr, CrPyr treatment group with 0.6 mL physiological saline (0.75%) containing 12 mg CrPyr per egg.

2) Pooled SEM (n = 8).

**Table 5:** Effects of *in ovo* feeding of creatine pyruvate on the concentration of creatine and phosphocreatine in thigh muscle of embryos and broilers on 2 d after injection, the day of hatch, 3, and 7 d post-hatch

Items	Treatment1)			SEM2)	p-value

	Control	Saline	CrPyr		
Cr (μmoL/g)					
2 d after injection	11.46^b^	10.99^b^	13.35^a^	0.36	0.012
Hatch	11.23^b^	10.98^b^	12.72^a^	0.29	0.026
3 d	15.52	15.46	16.48	0.21	0.085
7 d	19.01	17.56	17.97	0.28	0.097
PCr (μmoL/g)					
2 d after injection	0.60	0.58	0.67	0.02	0.117
Hatch	0.33	0.35	0.34	0.03	0.278
3 d	1.01	1.03	0.99	0.02	0.589
7 d	1.11	1.09	1.08	0.02	0.847

The results are presented by mean values and the SEM.

CrPyr, creatine pyruvate; SEM, standard error of the mean.

1) Control, non-injected control group; Saline, saline control group with 0.6 mL physiologicalsaline (0.75%) per egg; CrPyr, CrPyr treatment group with 0.6 mL physiological saline (0.75%) containing 12 mg CrPyr per egg.

2) Pooled SEM (n = 8).

a,b Means within a row with different superscripts are different at p<0.05.

**Table 6:** Effects of *in ovo* feeding of creatine pyruvate on the creatine kinase activity in thigh muscle of embryos and broilers on 2 d after injection, the day of hatch, 3, and 7 d post-hatch (U/mg of protein)

Item	Treatment1)			SEM2)	p-value

	Control	Saline	CrPyr		
2 d after injection	2.58^b^	2.68^b^	3.14^a^	0.10	0.004
Hatch	3.16^b^	3.30^b^	3.43^a^	0.04	0.012
3 d	3.95^b^	4.07^b^	4.13^a^	0.03	0.030
7 d	5.38	5.28	5.37	0.03	0.355

The results are presented by mean values and the SEM.

CrPyr, creatine pyruvate; SEM, standard error of the mean.

1) Control, non-injected control group; Saline, saline control group with 0.6 mL physiologicalsaline (0.75%) per egg; CrPyr, CrPyr treatment group with 0.6 mL physiological saline (0.75%) containing 12 mg CrPyr per egg.

2) Pooled SEM (n = 8).

a,b Means within a row with no common superscript differ significantly (p<0.05).

**Table 7:** Effects of *in ovo* feeding of creatine pyruvate on the hexokinase and pyruvate kinase activities in thigh muscle of embryos and broilers on 2 d after injection, the day of hatch, 3, and 7 d post-hatch

Items	Treatment1)			SEM2)	p-value

	Control	Saline	CrPyr		
HK (U/g of protein)					
2 d after injection	28.71	28.93	29.44	0.51	0.849
Hatch	25.02^b^	26.10^b^	32.42^a^	1.03	0.002
3 d	34.13	34.83	35.79	0.56	0.545
7 d	30.57	29.69	28.74	0.47	0.386
PK (U/g of protein)					
2 d after injection	426.93^b^	421.48^b^	468.99^a^	9.97	0.036
Hatch	428.54^b^	430.26^b^	461.85^a^	6.81	0.048
3 d	396.62^b^	375.14^b^	439.74^a^	10.53	0.007
7 d	342.73	345.46	357.25	4.67	0.425

The results are presented by mean values and the SEM.

CrPyr, creatine pyruvate; SEM, standard error of the mean; HK, hexokinase; PK, pyruvate kinase.

1) Control, non-injected control group; Saline, saline control group with 0.6 mL physiologicalsaline (0.75%) per egg; CrPyr, CrPyr treatment group with 0.6 mL physiological saline (0.75%) containing 12 mg CrPyr per egg.

2) Pooled SEM (n = 8).

a,b Means within a row with different superscripts are different at p<0.05.

**Table 8:** Effects of *in ovo* feeding of creatine pyruvate on the mRNA expressions of GLUT3, GLUT8 in thigh muscle of embryos and broilers on 2 d after injection, the day of hatch, 3, and 7 d post-hatch

Items	Treatment1)			SEM2)	p-value

	Control	Saline	CrPyr		
Relative expression of GLUT3					
2 d after injection	1.03	1.08	1.11	0.06	0.880
Hatch	1.03^b^	0.99^b^	1.30^a^	0.05	0.013
3 d	1.02	0.98	0.97	0.03	0.840
7 d	1.02	1.08	1.15	0.36	0.386
Relative expression of GLUT8					
2 d after injection	1.04	0.97	0.99	0.05	0.844
Hatch	1.03^b^	1.06^b^	2.24^a^	0.13	<0.001
3 d	0.99	1.05	0.96	0.03	0.484
7 d	1.03	0.99	1.06	0.29	0.626

The results are presented by mean values and the SEM.

CrPyr, creatine pyruvate; SEM, standard error of the mean; GLUT, glucose transporter.

1) Control, non-injected control group; Saline, saline control group with 0.6 mL physiologicalsaline (0.75%) per egg; CrPyr, CrPyr treatment group with 0.6 mL physiological saline (0.75%) containing 12 mg CrPyr per egg.

2) Pooled SEM (n = 8).

a,b Means within a row with different superscripts are different at p<0.05.

**Table 9:** Effects of *in ovo* feeding of creatine pyruvate on the mRNA expressions of glycogen synthase and glycogen phosphorylase in thigh muscle of embryos and broilers on 2 d after injection, the day of hatch, 3, and 7 d post-hatch

Items	Treatment1)			SEM2)	p-value

	Control	Saline	CrPyr		
Relative expression of glycogen synthase					
2 d after injection	1.03	0.95	1.02	0.04	0.660
Hatch	1.02	1.04	0.93	0.04	0.476
3 d	1.03	1.02	1.02	0.02	0.965
7 d	1.04	1.12	1.04	0.04	0.573
Relative expression of glycogen phosphorylase					
2 d after injection	1.03	0.96	1.10	0.05	0.547
Hatch	1.01	0.98	0.95	0.03	0.678
3 d	1.02	1.04	1.05	0.02	0.860
7 d	1.01	0.99	1.03	0.02	0.847

The results are presented by mean values and the SEM.

CrPyr, creatine pyruvate; SEM, standard error of the mean.

1) Control, non-injected control group; Saline, saline control group with 0.6 mL physiologicalsaline (0.75%) per egg; CrPyr, CrPyr treatment group with 0.6 mL physiological saline (0.75%) containing 12 mg CrPyr per egg.

2) Pooled SEM (n = 8).
